# Engaging older adults in self-management talk in healthcare encounters: a systematic review protocol

**DOI:** 10.1186/s13643-020-1276-1

**Published:** 2020-01-16

**Authors:** Michael T. Lawless, Phoebe Drioli-Phillips, Mandy M. Archibald, Alison L. Kitson

**Affiliations:** 1 Caring Futures Institute, College of Nursing and Health Sciences, Flinders University, Bedford Park, SA 5042 Australia; 2 National Health and Medical Research Council Transdisciplinary Centre of Research Excellence in Frailty Research to Achieve Healthy Ageing, Adelaide, SA Australia; 3 School of Psychology, University of Adelaide, Adelaide, 5005 Australia; 4 Helen Glass Centre for Nursing, University of Manitoba, 99 Curry Place, Winnipeg, Canada

**Keywords:** Aged, Frail elderly, Chronic illness and disease, Conversation analysis, Communication, Healthcare delivery, Self-management, Qualitative research, Protocol

## Abstract

**Background:**

Clinical practice guidelines for the management of complex chronic conditions in older adults encourage healthcare providers to engage patients in shared decision-making about self-management goals and actions. Yet, healthcare decision-making and communication for this population can pose significant challenges. As a result, healthcare professionals may struggle to help patients define and prioritise their values, goals, and preferences in ways that are clinically and personally meaningful, incorporating physical functioning and quality of life, when faced with numerous diagnostic and treatment alternatives. The aim of this systematic review is to locate and synthesise a body of fine-grained observational research on communication between professionals, older adults, and carers regarding self-management in audio/audio-visually recorded naturalistic interactions.

**Methods/design:**

The paper describes a systematic review of the published conversation analytic and discourse analytic research, using an aggregative thematic approach and following the PRISMA-P guidelines. This review will include studies reporting on adult patients (female or male) aged ≥ 60 years whose consultations are conducted in English in any healthcare setting and stakeholders involved in their care, e.g. general practitioners, nurses, allied health professionals, and family carers. We will search nine electronic databases and the grey literature and two independent reviewers will screen titles and abstracts to identify potential studies. Discrepancies will be resolved via consultation with the review team. The methodological quality of the final set of included studies will be appraised using the Joanna Briggs Institute Critical Appraisal Checklist for Qualitative Research and a detailed description of the characteristics of the included studies using a customised template.

**Discussion:**

This is the first systematic review to date to locate and synthesise the conversation analytic research on how healthcare professionals raise and pursue talk about self-management with older adults in routine clinical interactions. Amalgamating these findings will enable the identification of effective and potentially trainable communication practices for engaging older adults in healthcare decision-making about the self-management goals and actions that enable the greatest possible health and quality of life in older adulthood.

**Systematic review registration:**

PROSPERO CRD42019139376

## Background

Caring for older adults living with multimorbidity (defined as two or more chronic conditions) and/or related complex long-term conditions including frailty (hereafter older adults) is an increasingly common aspect of primary care practice. Yet, healthcare communication and decision-making for this group can pose considerable challenges. This is due to factors including (a) the need for healthcare professionals to consider the implications of multiple problems and medications within a single clinical encounter; (b) difficulties in identifying problems due to certain conditions being clinically dominant or highly symptomatic; (c) different conditions requiring separate, time-intensive treatment planning; and (d) a shortage of evidence describing interactions between and among combinations of conditions and treatments [1, 2]. As a result, healthcare professionals may struggle to help aged and frail patients define and prioritise their values, goals, and preferences in ways that are clinically relevant and personally meaningful when faced with numerous diagnostic and treatment alternatives [2–4]. Compliance with multiple disease-specific guidelines can lead to polypharmacy, high treatment burden, inattention to social and environmental context, and increase the risk that patients will receive fragmented care that is not always reflective of their goals and preferences, thereby threatening care quality and patient safety [5]. Difficulties with exploring patients’ perspectives, translating problems into goals, and making shared decisions about actions often mean that patients’ non-medical goals may be marginalised or under-acknowledged. This is especially true for older adults whose goals may be multi-dimensional, focusing not only on medical aspects, but also on psychosocial concerns such as caregiving-related stress, identity, changing social roles/relationships, sexual function, and financial management [6].

Healthcare delivery must be transformed to address the challenges of promoting shared decision-making with older adults with complex care needs [7, 8]. This entails a shift from disease-specific to patient-centred models of care [9]. To implement a patient-centred approach, healthcare providers are advised to elicit patients’ concerns, values, and preferences and centre the delivery of healthcare on these perspectives. This process can enable greater patient engagement in healthcare decision-making [10], greater self-management abilities, increased trust in healthcare professionals, and improved patient health outcomes, including on physiological measures, health status, and measures of functional ability [11]. Establishing patient goals is a widely recommended strategy for engaging patients with multimorbidity in shared decision-making, but there is little evidence to support its use in routine clinical practice [12–15]. Goal setting is typically defined as “a process by which healthcare professionals and patients agree on a health-related goal” [12]. Although this definition acknowledges the health-related goal setting in the context of behaviour change and action planning for chronic conditions, it does not explicate these interactional processes in relation to self-management communication and decision-making [1]. An explicit, structured, and patient-centred discussion of goals of care may not only more effectively engage individuals in care planning leading to better health and quality of life outcomes, but also contribute to the long-term accessibility, affordability, and quality of the healthcare system [15, 16].

How best to support shared decision-making for older adults living with multiple health and social care needs is an emerging focus of healthcare research, education, and policy [7, 10, 17]. A review of the communication practices involved in healthcare decision-making as it relates to self-management can help identify effective and potentially trainable strategies to improve older adults’ participation in decisions relevant to their health and care. An earlier systematic review by Land et al. [18] summarised the findings of 28 observational studies of healthcare interaction, mapping communication practices that encourage or constrain shared decision-making. A second systematic review undertaken by Albury et al. [19] thematically synthesised 10 studies of communication about health behaviour change in primary care. Both reviews offer valuable insights into the structure and elements of shared decision-making and health behaviour change communication sequences. Nonetheless, the structure and communication practices identified in these reviews are not specific to encounters involving older adults. Furthermore, the earlier reviews were not focused on shared decision-making processes as they relate to different aspects of self-management, which encompasses not only behavioural modification and/or lifestyle adaptation, but also chronic disease management, social support, resource management, and psychological and stress management [20]. A preliminary search of PROSPERO, MEDLINE (via Ovid), the Cochrane Database of Systematic Reviews, and the Joanna Briggs Institute (JBI) Database of Systematic Reviews and Implementation Reports failed to locate a systematic review (neither published nor in progress) on communication practices specific to older adults’ engagement in self-management discussions in clinical encounters. We therefore aim to synthesise evidence from conversation analysis (CA) and discourse analysis studies of healthcare communication between professionals, older adults, and carers regarding self-management in audio/audio-visually recorded naturalistic interactions.

## Methods/design

This systematic review protocol has been registered with the International Prospective Register of Systematic Reviews (PROSPERO; Protocol ID: CRD42019139376). The Preferred Reporting Items for Systematic Review and Meta-Analysis Protocols 2015 checklist (PRISMA-P) was used to develop this protocol (see Additional file 1). Approaches to data analysis and the presentation of results in CA research differ from conventional qualitative research. We will therefore follow established methodological recommendations for systematically reviewing and appraising CA evidence [21] and adhere to the enhancing transparency in reporting the synthesis of qualitative research (ENTREQ) guidelines [22].

### Study design

This review will employ an aggregative thematic approach [23] to synthesise the findings of the studies in accordance with contemporary recommendations for CA evidence syntheses [21]. We chose this inductive and iterative approach as it allows for “themes” (i.e. major categories of communication practices and their sequential positioning) to emerge from the data and common attributes/functions to be identified. The six-stage approach of systematically reviewing and synthesising evidence from CA studies includes (1) articulating the purpose, audience, review question(s), and scope; (2) specifying eligibility criteria; (3) searching for studies; (4) describing characteristics of the included studies (including quality appraisal); (5) data extraction; and (6) collating and synthesising the data.

### Study aim

#### Review questions

1. What published research exists on how healthcare professionals initiate, address, and/or pursue talk about self-management with older adults and/or their carers in healthcare encounters?
2. Which patient/carer actions contribute to older adults’ participation in decisions relevant to self-management? Participation includes providing older adults opportunities to voice concerns, set or negotiate goals, make action plans, and/or orient to or accept healthcare professionals’ proposals for future action.
3. How do healthcare professionals’ communication practices enable or constrain patient participation in decision-making relevant to self-management goals and actions?
4. What are the opportunities to inform healthcare communication policy, practice, and/or training on how to provide older adults with opportunities to participate in decision-making regarding their health, wellbeing, and care?

### Inclusion criteria

A modified version of the SPIDER (Sample, Phenomenon of Interest, Design, Evaluation, Research type) formula for qualitative research will be used in lieu of the PICO (Population, Intervention, Comparison, Outcome) framework to help formulate the research questions and conceptualise the eligibility criteria for this review [24]. The SPIDER framework, originally proposed as a method for qualitative and mixed methods evidence retrieval, was selected due to its greater specificity in identifying the relevant CA evidence when compared to PICO [25].

### Participants

This review will consider peer-reviewed studies reporting on adult patients (female or male) aged ≥ 60 years whose consultations are conducted in English in any healthcare setting and stakeholders involved in their care, e.g. general practitioners, nurses, and family carers. No limitation will be placed on the upper age, gender, healthcare professionals’ roles, ethnicity, or geographical location of patients, or the number or types of health conditions. Studies reporting on younger individuals (i.e. aged < 60 years) will be considered if the median age of participants is ≥ 60 years and individual data extracts can be linked to the older participants. We will not include studies reporting on patients with dementia or those receiving palliative/end-of-life care only given the distinct considerations for healthcare communication and decision-making for these populations.

### Condition

The condition(s)/domain(s) being studied in this review are chronic conditions, multimorbidity, and complex chronic conditions, defined as the co-occurrence of chronic conditions affecting three or more body systems in one person, without a defining indexing condition [26]. These conditions being common among older adults where addressing risk and modifiable factors, eliciting perspectives/concerns, setting goals, discussing options, and developing care plans are relevant to healthcare communication, decision-making, and provision.

### Intervention/phenomenon of interest

We conceptualised the intervention/phenomenon of interest as the provision of opportunity for healthcare decision-making regarding self-management issues/behaviours, including medical, psychosocial, and/or behavioural prevention or risk-management issues/behaviours, relevant to complex chronic conditions in older adulthood in the context of routine healthcare encounters in any setting. Comparators and/or controls are not applicable to this review.

For the purpose of this review, we define “self-management talk” as talk that participants observably treat as focusing on, or relevant to, chronic disease management, including patient-actioned or healthcare professional-actioned medical and/or behavioural prevention; self-management of medical, functional, social, and/or emotional issues; and/or risk-management behaviours/issues. It includes communication that references states, events, and/or actions:

1. In the domain of human activity and individual persons (i.e. physician-patient interaction/relations in healthcare settings as opposed to, e.g. cellular communication and computer communication networks);
2. That may or will occur in relation to individual persons and are, in this context, negative or potentially negative; amenable or potentially amenable to behavioural/medical prevention or management;
3. That may be certain or uncertain;
4. That may or will happen after the present interactional episode;
5. That includes communication about concerns, feelings/emotional responses to concerns (including medical and non-medical concerns), goals, and action plans; and
6. That is not exclusively focused on eliciting concerns or setting a future course of action in relation to existing problems.

### Outcomes

#### Primary outcomes

The outcomes of this review relate to the incidence, structure, and local consequences of self-management talk (i.e. how talk is raised, addressed, and/or pursued) and older adults’ participation in healthcare decision-making (e.g. setting goals and actions). Reflecting our agnostic stance towards the normativity of providing opportunities for patients to participate in decision-making in routine practice, the primary outcomes are as follows:

1. The identified communication practice(s) provide(s) healthcare professionals or service users (older adults and/or carers) with opportunities to pursue talk about or relevant to self-management, including medical or behavioural (healthcare provider-led or patient-led) health prevention and risk-management behaviours/issues.
2. The identified communication practice(s) provide(s) service users with opportunities to participate in healthcare decision-making relevant to self-management issues and/or behaviours. “Participation” will be defined in terms of commitment points in typical healthcare encounters (e.g. actions prior to a decision being broached, proposing a future course of action, and committing/withholding commitment to the proposed course of action) and specifically places in turns at talk when it becomes relevant for patients to contribute to healthcare decision-making, e.g. eliciting perspectives/concerns, agreeing on goals, negotiating options, and/or committing or not to a proposed or implicated future course of action.

### Context

We will search for studies focusing on talk relevant to healthcare both in professional (e.g. clinics, general practice surgeries, counselling, and helplines) and informal (e.g. telephone calls) settings, if applicable.

### Types of studies

We will include peer-reviewed empirical research (i.e. not conference presentations or graduate theses) reporting on fine-grained analysis of audio/audio-visually recorded, naturally occurring (i.e. interactions that would have occurred whether or not the research was undertaken) healthcare consultations in English. CA studies and discourse analysis studies involving qualitative interactional analysis of authentic communication episodes will be eligible for inclusion. Restrictions are eligible studies must include collection and fine-grained analysis of audio and/or visual recordings of actual interpersonal communication episodes with co-present older adults and healthcare professionals.

### Search strategy

The search strategy aims to find published peer-reviewed empirical studies. All searches, including grey literature searches, will be restricted to studies in English for practical reasons and due to the possibility that different languages might entail divergent practices for talking about self-management as well as different interactional consequences. No restriction will be placed on publication dates.

This review will use a three-step search strategy. First, in conjunction with a university research librarian, we will undertake a preliminary limited search of PubMed and CINAHL, followed by a structured analysis of text words contained in titles and abstracts and of the index terms used in the article descriptions. This initial step will ensure that the search strategy is sufficiently sensitive, precise, and specific with respect to our research objectives and the population, concepts, and context of interest in this review. Second, we will undertake a structured search across all included electronic databases using the identified keywords and index terms. Third, the reference lists of all included studies will be searched manually for additional eligible sources.

The electronic databases to be searched include the following:

- AMED
- ASSIA
- CINAHL
- EMBASE
- PubMed
- PsycINFO
- Scopus
- ISI Web of Science
- Sociological Abstracts CSA

Other sources include the following:

- Grey literature searching (Open Grey, Australian National Library, Mednar, Grey Literature Report, Grey Literature Network)
- Specialist online bibliographies (e.g. EM/CA Wiki [emcawiki.net])
- Our own and other academics’ personal reference collections
- Citation tracking
- Reference list searching

Initial keywords to be used will be “communicat*” OR “interact*”, “aged*” OR “older*”, and “conversation analy*”. Keywords and Medical Subject Heading (MeSH) terms pertinent to communication, conversation analysis, self-management, and older adults will be identified and optimised (Table 1). A provisional search strategy will be designed for PubMed and adapted to suit individual databases (see Additional file 2). The full search strategy will be reported in the follow-up publication.

**Table 1:** Search terms using SPIDER headings

SPIDER heading	Search terms for PubMed database
Sample: adults (male of female) aged ≥ 60 years	“Aged[mh]” OR “frail elderly[mh]” OR “older[tw]” OR “elder[tw]” OR “geriatric[tw]” AND
Phenomenon of interest: communication about self-management in clinical encounters	“communication[mh]” OR “interpersonal relations[mh]” OR “interaction[tw]” OR “personal communication[tw]” AND “self-management[mh]” OR “self care[mh]” OR “patient care planning[mh]” OR “care plan[tw]” OR “directive counselling[mh]” OR “health coaching[tw]” OR “motivational interviewing[mh]” OR “healthy lifestyle[mh]” OR “goal setting[tw]” AND “clinical[tw]” OR “referral and consultation[mh]” OR “medical[tw]” OR “health[tw]” AND
Design: qualitative research	“qualitative research[mh]” OR “qualitative study[tw]” OR “linguistic analysis[tw]” OR “sequential analysis[tw]” AND
Evaluation: opportunity to discuss self-management, participation in healthcare decision making	“communication[mh]” OR “interpersonal relations[mh]” OR “interaction[tw]” OR “personal communication[tw]” AND
Research type: conversation analysis and related discursive research	“conversation analysis[tw]” OR “sequential analysis[tw]” OR “discourse analysis[mh]” OR “discursive psychology[tw]” OR “linguistic analysis[tw]” OR “membership categorisation analysis[tw]”

### Study selection

Two members of the review team will conduct the initial literature search. Following the search, the identified studies will be uploaded into EndNote X7 (Clarivate Analytics, PA, USA). Studies will be selected via a two-step process involving (1) initial scanning of titles and abstracts by two independent reviewers using a standardised screening form and (2) full-text review of potentially relevant articles by the same reviewers. Endnote X7 will be used to store and manage the downloaded bibliographic information. Covidence systematic review software (Veritas Health Innovation, Melbourne, Australia) will be used to remove duplicates, conduct screening, and securely store data for future updates of the review [27]. Disagreement about eligibility between the two reviewers will be resolved through discussion. In cases of uncertainty about eligibility, a third reviewer will be consulted to assist with the final selection of studies. We will organise regular meetings for the duration of the project to report on progress and discuss emerging findings. Results of the search will be presented in a PRISMA flow diagram.

### Data extraction

We will use a standardised data extraction form, adapting category labels developed for an earlier systematic review of CA studies [21] while referring to contemporary guidance on data extraction in systematic reviews of qualitative evidence [28]. Data from the included studies will be extracted by two reviewers and then compared to maintain uniformity during the extraction processes. Data extraction will be limited to, and focused on, the review questions and include details about aims, participant characteristics, data characteristics, analysis characteristics, findings related to communication practices, and their function with respect to raising and pursuing self-management talk and/or encouraging patient participation in decision-making. Authors of primary studies may be contacted for clarification or missing information if necessary. The variables to be included in the data extraction form are reported in Table 2.

**Table 2:** Data extraction form

Author and date	
• Bibliographic details: o Title of study o Author(s) o Year of publication o Source (e.g. journal title, volume, and issue; book reference and pp.) • Country of study • Academic field of publication (e.g. sociology, medicine) • Aim of the study • Addition research questions/objectives • Study design characteristics: o Methodological approach (as described by the author(s)) • Setting: o Number of sites (e.g. two sites of the same setting, e.g. two general practice clinics) o Number of institutional settings • Participant characteristics (N, age, health status/conditions, other reported information) • Data characteristics: o Recording methods (audio, audio-visual) o Size of the overall dataset (minutes; number of interactions recorded) o Number of reported episodes (extracts)—number of episodes in the data collection/corpus pertaining to EACH finding o Are interactions one-on-one, multiparty, or both? o Institutional or mundane interaction or both? o Is the practice observed in more than one group? (do patients or providers use it, or both?) • Analysis characteristics—does the analysis: o Examine interactional data in fine-grained detail? o Attend to sequence (i.e. examine more than one party’s turns)? o Examine more than topical/semantic content? (i.e. does it examine aspects of grammar, syntax, pragmatics, and/or prosody?) o Includes examination of interactional consequences/outcomes? o Includes deviant cases? o Support central analytic claims by direct references to the data (e.g. quotations, extracts)? (no/occasionally/predominantly) o Are key analytic claims supported by reference to relevant published sources? (no/occasionally/predominantly) • Findings reported (broad)—complete for each study: o Results summary (e.g. as reported in the abstract) o Number of relevant primary findings (types of practices) o Number of relevant secondary findings (types of practices) • Findings reported (narrow)—complete for each identified phenomenon o Phenomenon (i.e. type of communication practice, in brief) o Phenomenon in the author’s own words o Related research question (if stated) o Number of episodes (extracts) pertaining to this finding in the article o Typical/archetypal sequence—include direct example(s) o Sequence/turn design features of the phenomenon o Interactional effects of the design features o Overall function of the phenomenon o Proposed implications in author’s own words o Other possible implications (reviewer’s comment) • Conclusions • Study limitations • Author recommendations	

### Assessment of methodological quality

Two independent reviewers will use an adapted version of the JBI Critical Appraisal Checklist for Qualitative Research [28] to appraise the methodological quality of the included studies. All modifications to or deviations from the appraisal criteria will be reported, justified, and attached as an appendix/additional file in the follow-up publication. Any disagreements that may arise among the reviewers will be resolved via discussion and/or consultation with a third reviewer. All studies, irrespective of the results of the methodological quality assessment, will undergo data extraction and synthesis. The quality of the selected studies will be considered during data extraction and analysis and will for this reason be reflected in the results and conclusion of this systematic review [21].

### Data synthesis

We will synthesise the findings of the included studies using an aggregative thematic approach used previously for the collation of CA evidence [21]. The data synthesis procedure will involve the following iterative steps: (1) repeatedly reading the completed extraction forms to enable familiarity with the data; (2) sorting the studies into logical/meaningful categories; (3) organising and amalgamating the findings of the studies on the basis of similarity of discursive and linguistic construction, subject positioning, and/or interactional function(s); (4) continually referencing the literature to explicate and cross-examine identified communication practices; (5) consulting with a local advisory group comprising clinicians and service users to ensure relevance to current clinical practice and alignment with stakeholder priorities; (6) identifying notable evidence gaps; and (7) deriving and discussing implications for potential audience(s). Charts and tables will be used, where appropriate, to map and summarise the relevant publication characteristics and findings related to the communication practices, their functions, and the settings in which they were documented. Analysis of subgroups and/or subsets is not planned.

### Assessing certainty in the findings

We will grade the final synthesised findings according to the ConQual approach for establishing confidence in the outputs of qualitative syntheses [29]. A summary of findings table will chart the major elements of the review (e.g. study population characteristics, communication phenomena of interest, and healthcare context), report the final ConQual scores, and detail how and when these scores were derived. We will also weave commentary on the quality of the evidence for each finding into the synthesis. This measure will help overcome the difficulties that may arise when attempting to applying the ConQual ranking system to CA evidence.

## Discussion

A comprehensive synthesis of primary CA research on healthcare communication between health professionals, older adults, and carers regarding self-management goals and actions is lacking. Developing a systematic approach to the quality and timing of communication about care goals is a low-risk, high-value intervention for promoting older adults’ engagement in self-management shared decision-making. This review was therefore designed to develop a more detailed understanding of (1) how healthcare professionals in primary care communicate with older adults about goal-setting in relation to long-term condition management, (2) which communication techniques are most likely to be effective in principle, and (3) which practices cause communication difficulties or breakdown.

To ensure relevance to local clinical practice and facilitate wider knowledge translation, the review findings will be shared and discussed with healthcare professionals, clinical educators, and service users at scheduled intervals through a series of consultative and collaborative meetings. Academic dissemination will occur through peer-reviewed publication and presentation at various public forums and conferences across disciplines. The results of this review will directly inform the next phase of a multi-phase knowledge translation project that aims to investigate communication problems and solutions in routine clinical encounters to improve engagement, involvement, and quality of care.

## Strengths and limitations

This review will use observable participant responses (verbal and non-verbal) as proxies for the effectiveness of specific conversational practices in relation to patient and/or carer outcomes such as physical health status (including clinical outcomes and patient-reported physical health), psychological and psychosocial health status (including quality of life), health behaviours, and/or treatment burden [30]. Additionally, this study will only consider studies reporting on interactions in English. Studies on non-English languages could provide valuable insights and conceptualisations and should be considered in future reviews of healthcare interactions involving diverse cultural and linguistic groups.

A major strength of this review will be its use of transparent and systematic methods to synthesise CA research on self-management talk with a focus on identifying effective and potentially trainable strategies for promoting older adults’ involvement in communication and decision-making processes relevant to their healthcare. The involvement of multiple independent reviewers to compare and cross-examine concepts, types and descriptions of conversational practices, interpretations and overall reviewer findings and critical appraisal will strengthen the quality of the review. These measures will ensure that the synthesis findings and recommendations are robust, well-developed, and comprehensive, bolstering the trustworthiness of the review findings and applicability to clinical education and practice.

## Supplementary information

**Additional file 1.** Preferred Reporting Items for Systematic Review and Meta-Analysis Protocols (PRISMA-P) 2015 checklist.

**Additional file 2.** PubMed search strategy.

## Data Availability

Not applicable

## Abbreviations

AMED: Allied and Complementary Medicine Database
ASSIA: Applied Social Sciences Index and Abstracts
CA: Conversation analysis
CINAHL: The Cumulative Index to Nursing and Allied Health Literature
EM/CA: Ethnomethodology/conversation analysis
ENTREQ: Enhancing transparency in reporting the synthesis of qualitative research
PICO: Population, Intervention, Comparison, Outcome
PRISMA-P: Preferred Reporting Items for Systematic Review and Meta-Analysis Protocols
PROSPERO: International Prospective Register of Systematic Reviews
SPIDER: Sample, Phenomenon of Interest, Design, Evaluation, Research type
