# Supplementary material for: Engaging older adults in self-management talk in healthcare encounters: a systematic review protocol
Source: Syst Rev. 2020 Jan 16;9:15. doi: 10.1186/s13643-020-1276-1 (PMC6964206; doi:10.1186/s13643-020-1276-1)
Supplement: Supplementary file 2 — Additional file 2. PubMed search strategy. [file 13643_2020_1276_MOESM2_ESM.docx]

**Additional file 2.** PubMed search strategy.

(((((("Aged"[mh] OR "Aged"[majr] OR "Aged, 80 and over"[mh] OR "Aged, 80 and over"[majr] OR "frail elderly"[mh] OR "frail elderly"[majr] OR older*[tw] OR elder*[tw] OR geriatric patients[tw] OR geriatric*[tw])) AND

("communication"[mh] OR "interpersonal relations"[mh] OR "interpersonal relations"[majr] OR interact*[tw] OR personal communicat*[tw])) AND ("self-management"[mh] OR "self-management"[majr] OR "self care"[mh] OR "self care"[majr] OR "clinical decision making"[mh] OR "clinical decision making"[majr] OR "directive counseling"[mh] OR "directive counseling"[majr] OR "patient care planning"[mh] OR "patient care planning"[majr] OR "care plan*"[tw] OR "healthy lifestyle"[mh] OR "healthy lifestyle"[majr] OR "risk reduction behavior"[mh] OR "risk reduction behavior"[majr] OR risk reduction*[tw] OR "motivational interviewing"[mh] OR "motivational interviewing"[majr] OR "Goals"[mh] OR "Goals"[majr] OR "goal setting"[tw])) AND

(Clinical*[tiab] OR "referral and consultation"[mh] OR "referral and consultation"[majr] OR Medical*[tw] OR Health*[tw])) AND

(discourse analysis[tw] OR conversation analy*[tw] OR sequential analy*[tw] OR discourse analy*[tw] OR discursive psychol*[tw] OR linguistic analy*[tw] OR membership category action analy*[tw])) AND

English[Language] AND

(Humans[Mesh)]

No limitation on publication dates
